# Double-spiral as a bio-inspired functional element in engineering design

**DOI:** 10.1038/s41598-024-79630-6

**Published:** 2024-11-25

**Authors:** Mohsen Jafarpour, Mohammad Aryayi, Stanislav N. Gorb, Hamed Rajabi

**Affiliations:** 1 Functional Morphology and Biomechanics, Institute of Zoology, Kiel University, 24118 Kiel, Germany; 2 University of Burgundy, 21000 Dijon, France; 3 Division of Mechanical Engineering and Design, School of Engineering, London South Bank University, London, SE1 0AA UK; 4 Mechanical Intelligence Research Group, School of Engineering, London South Bank University, London, SE1 0AA UK

**Keywords:** Biomimetics, Bio-inspired design, Adaptive structures, 3D printing, Soft robotics, Mechanical engineering, Soft materials

## Abstract

Spiral, one of the most well-known functional patterns in nature that can be observed in structures such as the proboscis of lepidoptera and snail shells or as vortices forming in flowing fluids, has long served as a source of inspiration for humans in the creation of numerous spiral-based designs. Double-spiral is a design derived from spirals, which has been previously presented and utilized as a compliant joint. Advantageous properties of double-spirals, such as easily adjustable design, multiple degrees of freedom, reversible extensibility, and tunable deformability make them promising candidates for the development of mechanically intelligent structures that exhibit unique behavior and reach desired functions, such as soft grippers, continuum manipulators, energy-dissipative structures, and foldable metamaterials. In this article, we first develop the Double-Spiral Design software to facilitate the design and modeling of double-spirals. We then design and manufacture five different spiral-based structures using three-dimensional (3D) printing, including (1) a freeform passive gripper, (2) a highly extensible enveloping gripper, (3) a mechanical interlocking structure, (4) an adaptive energy-dissipative structure, and (5) a compliant planar joint. Through practical experimentation, we test the functionality of the developed structures and showcase the potential of double-spirals for being used in various technical applications. This study represents a significant step towards a better understanding of double-spirals and demonstrates their broad but unexplored potential in engineering design.

## Introduction

Bio-inspired design encompasses an interdisciplinary approach that utilizes nature’s strategies to develop advanced technological solutions. By adopting or deriving insights from biological structures, we can design and manufacture products that are more efficient and durable^1–5^. Although fundamental differences between natural and technological systems cause numerous difficulties in the implementation of biologically-inspired solutions into real technological applications, this approach has been increasingly applied in various technical fields.

Biomimetics usually requires an abstraction step rather than a simple and direct copying approach. Engineers often try to modify or simplify complex natural designs to suit human needs and overcome manufacturing limitations. Sometimes they can mimic only one aspect of a biological system and develop a solution for a specific engineering problem, but sometimes they use a concept inspired from the nature for a broad range of totally different purposes^1–4^.

Spiral, as an omnipresent geometrical pattern in nature, serves as a profound source of inspiration for humans^6,7^. Engineers across various disciplines have leveraged the spiral pattern, each offering their unique perspective, modifying its structure, and adapting it to meet requirements of specific applications. Spiral has also inspired the development of numerous engineering structures and systems, including flexible electronics^8,9^, springs^10–12^, joints^13,14^, metamaterials^15–18^, wearable thermoelectric generators^19^, soft actuators^20–24^, antennas^25^, soft grippers^26,27^, and heat exchangers^28,29^.

Compliant double-spirals have been presented recently as novel spiral-based structures^14^. They are inspired by the coiling-uncoiling behavior observed in deformable natural spirals, such as the arms of octopuses^30^, tail of chameleons^31^, tendrils of plants^32^, among others^33–36^. Double-spirals hold significant potential in practical applications due to their distinctive behavior when subjected to loading^14^. These structures offer the ability to undergo substantial and predictable deformations, making them particularly valuable in stretchable electronics, soft robots, or biomedical engineering devices where large deformations are necessary^37–39^.

Double-spirals, with their planar design and uniform cross-sectional profile along their depth, can be fabricated using various manufacturing methods, including additive, subtractive, and formative techniques^12–16^. As different additive manufacturing (3D printing) technologies have become increasingly widespread, fused deposition modeling (FDM) 3D printers are known for being a simple, low-cost, and easily accessible tool, suitable for rapid prototyping of parts using a wide variety of thermoplastic filaments with desired mechanical properties^40,41^. Consequently, researchers nowadays take advantage of computer-aided design and 3D printing to develop complex structures with enhanced functionalities, and soft robots are a prominent example of such advanced technical structures being rapidly developed through these methods^42–45^.

In the context of 3D printing soft robots, thermoplastic polyurethane (TPU) is a widely adopted material, alongside silicone elastomers, hydrogels, shape memory polymers (SMPs), and other thermoplastics, each offering unique characteristics^42–45^. TPU is well known for its high elasticity, flexibility, mechanical strength, and durability, making it ideal for creating components that require both stability and resilience^46–48^. It can withstand repeated mechanical stress while maintaining its shape and functionality, a crucial feature for soft robots, as these applications often involve frequent deformation and require resistance to fatigue in dynamic environments^49,50^. These properties, combined with its compatibility with various 3D printing techniques, make TPU a popular choice for a wide range of engineering applications^48^.

The purpose of this study is to demonstrate the potential of double-spirals fabricated with TPU as mechanical elements suitable for incorporation into a wide range of meticulously designed structures with various technical applications. It is anticipated that precise control over the geometry-function relationship in double-spirals will enable the development of structures with the desired stability and load-bearing capacity, while also benefiting from their high extensibility, adaptability, and durability.

Herein, we first develop a software package with a graphical user interface (GUI) to provide a user-friendly tool for designing double-spirals with different geometries. We then use the data exported from this software to make 3D models of spiral-based designs. Using a 3D printer, we manufacture them and investigate their mechanical behavior under different quasi-static loadings. Our results show that structures developed using compliant double-spirals are great examples of engineered designs that harness the mechanical compliance of their constituent elements (double-spirals here) to create motion when force is applied and achieve desired functionalities. By demonstrating the performance of double-spirals in different structures, this study proves their potential in further technical applications.

## Double-spiral design software

**Fig. 1:**
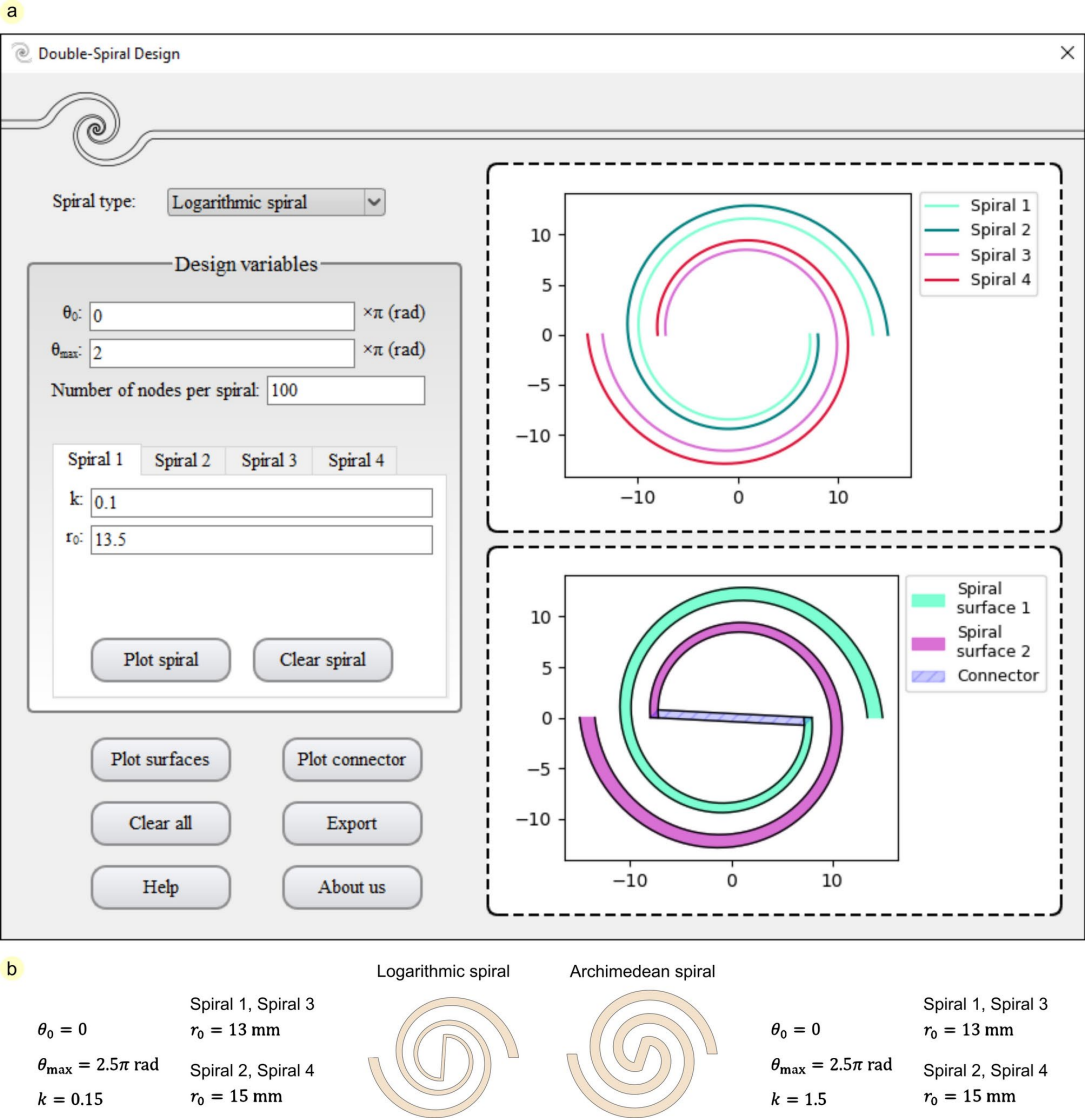
Double-Spiral Design software. (**a**) User interface of the software. After choosing between the logarithmic and Archimedean spirals, the user can define design variables for the spiral curves 1–4 to plot spiral surfaces 1 and 2. The desired double-spiral can be developed by plotting the connector. The data of all spiral curves and surfaces can be exported from the software in different formats. (**b**) An example of each logarithmic and Archimedean double-spirals and their corresponding design variables.

Herein, a software package, called Double-Spiral Design, was developed using Python scripting to facilitate the design and modeling of double-spirals (see Supplementary data). Thanks to mathematicians, the development of spirals with significantly different shapes using simple mathematical equations has facilitated their application in technical designs. Various two- and three-dimensional spirals, such as Archimedean, hyperbolic, parabolic, Galilean, lituus, logarithmic, Fibonacci, Cornu, helix, helicoid, and non-smooth spirals, are categorized and formulated for use^7^. In our software, either logarithmic or Archimedean spiral curves can be used to develop double-spirals with desired geometries (Fig. 1). The ability to choose between the logarithmic and Archimedean spirals facilitates the design of double-spirals with diverse geometries. While a double-spiral consisting of logarithmic spiral curves has a variable thickness, Archimedean spiral curves form a double-spiral with almost constant thickness (Fig. 1b). Equations (1) and (2) were used to define the logarithmic and Archimedean spirals in the polar coordinate system, respectively:

1 $$r = r_{0} e^{ - k\theta }$$

In this equation, $r_{0}$ is the radius of the spiral at $\theta = 0$, and $k$ is the polar slope.

2 $$r = r_{0} - k\theta$$

Here $r_{0}$ is the radius of the spiral at $\theta = 0$, and $k$ is the variation of the radius of the spiral with a rotation equal to one radian^7^.

The software uses the values of the design variables as inputs to plot four spiral curves (Fig. 1a). Plotting spiral curves 1 and 2 forms spiral surface 1, whereas plotting spiral curves 3 and 4, which are rotated about the origin of the coordinate system by 180°, forms spiral surface 2. The user can then ask the software to connect the inner ends of the plotted spiral surfaces to each other by two straight lines as a connector and accomplish the cross-sectional profile of a double-spiral model. Finally, the Double-Spiral Design software enables the user to save all plotted curves and surfaces in different formats (.csv, .dxf, .xlsx, .html, .txt, .step, and .stl).

## Spiral-based structures

We imported the data from the Double-Spiral Design software to the Abaqus software v. 6.14 (SIMULIA), developed five spiral-based structures, all consisting of logarithmic spiral curves (.stl files available as Supplementary data), and tested their performance in practice (Table 1). We used an FDM 3D printer (Prusa i3 MK3S, Prusa Research, Praha, Czech Republic) and a TPU filament (Flexfill TPU 98A, Fillamentum addi(c)tive polymers, Czech Republic) to manufacture the models. Highly durable TPU filament was used to ensure the large reversible deformability of the developed structures to meet the intended functions^14^. Fixtures and all other parts required for the experiments were printed with a polylactic acid (PLA) filament (Prusa Research, Praha, Czech Republic). The settings defined for 3D printing and the design variables of the developed double-spirals are given in Table S1 and Table S2, respectively. The mechanical behavior of the developed structures was characterized using a ZwickiLine uniaxial testing machine (Zwick Roell, Ulm, Germany) equipped with a 500 N load cell (Xforce P load cell, Zwick Roell, Ulm, Germany).

**Table 1:** Spiral-based structures developed in this study, their specifications, and the desired characteristics.

Spiral-based structure	Design specification	Desired characteristic
Freeform passive gripper	A chain of double-spirals in the form of a loop	(i) Local deformation (ii) Large, accumulated deformation
Highly extensible enveloping gripper	Six double-spirals forming a triangular structure	(i) High extension (ii) Inherent compliance
Mechanical interlocking structure	Double-spirals consisting of two geometrically different spiral coils	(i) Asymmetric rotation
Adaptive energy-dissipative structure	A series of double-spirals with different thicknesses	(i) Adaptive stiffness (ii) High extension
Compliant planar joint	Double-spirals with low extrusion height	(i) In- and out-of-plane deformation

In order to ensure the consistency of the results presented in this study, all experiments were conducted multiple times, and the average values were reported. Although any changes in the initial shape and functionality of the structures after experiments were negligible, an idling time of up to five minutes was provided for the structures between tests. Given the time-dependent behavior of TPU both under load and after load removal^51–53^, this idling time allowed for shape recovery and helped reduce the variability in results due to the viscous behavior of the material (see Discussion, paragraph on the mechanical behavior of TPU and Fig. 7).

### Freeform passive gripper

**Fig. 2:**
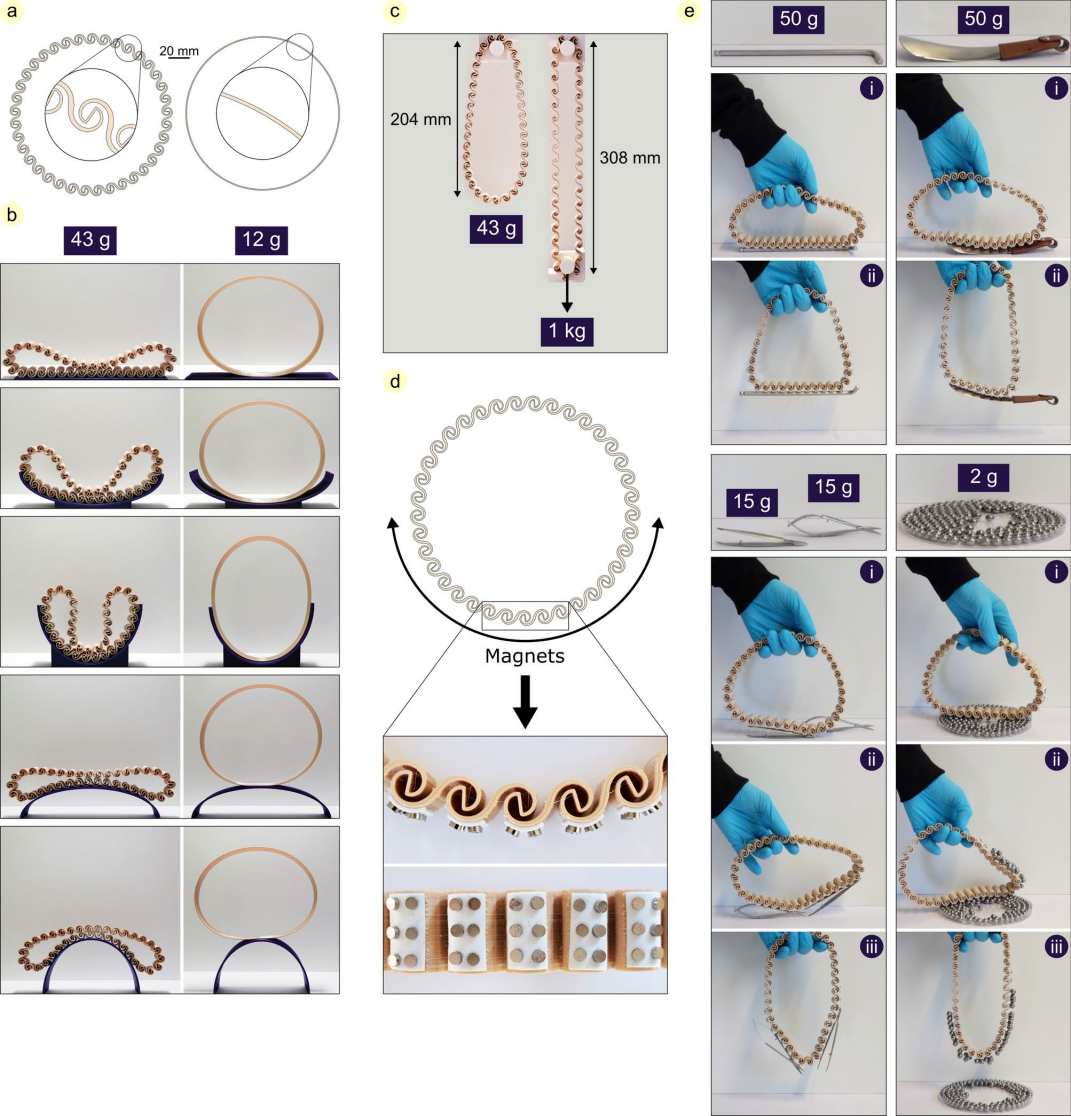
Freeform passive gripper. (**a**) 2D view of the double-spiral and circular loops. (**b**) Deformation of the loops under their own weight in contact with a flat, two concave, and two convex surfaces. (**c**) Deformation of the hung double-spiral loop under its own weight and a 1 kg mass. (**d**) Attaching magnets to the lower half of the double-spiral loop, with a close-up of the loop equipped with tiny disc magnets using double-sided tape, shown from both top and side views. (**e**) Using the developed magnetic gripper to pick up a 50 g Allen wrench, a 50 g shoehorn, two 15 g tweezers, and multiple 2 g steel beads.

Taking advantage of high rotational deformability of a chain of double-spirals, we designed a structure with enhanced adaptability. We connected a set of double-spirals with similar geometries to form a loop (Fig. 2a). The double-spiral loop deforms easily because each double-spiral can deform locally. In other words, it perfectly conforms to objects of various shapes due to the inherent deformability of each double-spiral. To clarify this feature, we designed another loop with the same diameter and 1 mm thickness (the average thickness of double-spirals), but having no double-spiral, and compared their deformability. We brought them in contact with five substrates with distinct curvatures: a flat surface, two concave, and two convex surfaces with a small and a large curvature. The structures were released to conform to the substrates due to their own weight (Fig. 2b). In all cases, the double-spiral loop exhibited a higher compliance and conformity to the substrates than the simple loop having no double-spiral.

In contrast to its high compliancy in bending and compression, the double-spiral loop exhibits increased stiffness when subjected to tensile forces, allowing it to withstand considerable tensile loads (Fig. 2c). These characteristics are particularly advantageous when the structure is employed as a gripper, enabling it on the one hand to efficiently adapt to objects of various geometries and on the other hand to endure their weights. Two principles of (i) the multichain structure and (ii) being compliant in compression while strong in tension, are well-known from insects’ tarsi^54–56^ and biological attachment devices^57–60^, respectively.

To demonstrate the functionality of this design—the ability to easily conform to different shapes (Fig. 2b) and to withstand tensile loads (Fig. 2c)—we added small magnets (Neodymium Rare Earth Magnets, 3 × 1.5 mm) to the outer surface of the double-spiral loop and made a magnetic gripper. Around 100 magnets (≈ 10 g altogether) were attached to the double-spirals of the lower half of the loop using double-sided tape (Fig. 2d), with their weight being negligible and not affecting the overall shape of the loop. We tested the performance of the developed gripper by picking up five randomly selected objects of various shapes and masses: a 50 g Allen wrench, a 50 g shoehorn, two 15 g tweezers, and multiple 2 g steel beads (Fig. 2e, Video S1). Approaching and conforming to the objects, followed by successfully picking them up, illustrates that the defined functions are achievable and proves the validity of the presented design.

### Highly extensible enveloping gripper

**Fig. 3:**
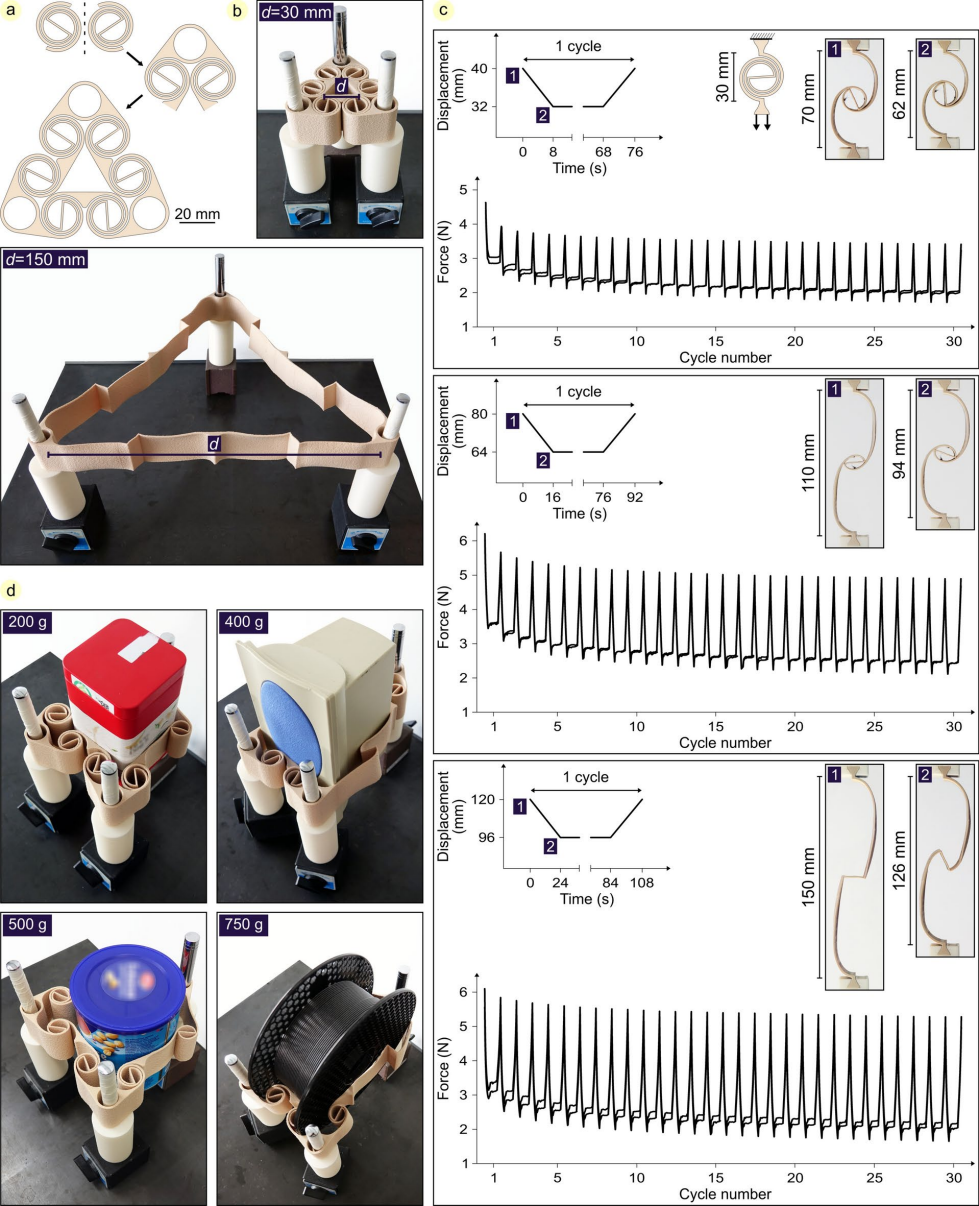
Highly extensible enveloping gripper. (**a**) Modelling the three-jaw gripper using three couples of symmetric double-spirals. (**b**) The 3D printed gripper placed on three legs in its initial and extended state. (**c**) Quantifying the mechanical behavior of two 3D printed double-spirals under a cyclic loading regarding their function in the gripper. (**d**) Gripping a 200 g cubic can, a 400 g loudspeaker, a 500 g cylinder can, and a 750 g filament spool using the developed spiral-based gripper.

Here, we take advantage of the high extensibility and inherent compliance of double-spirals. We designed and manufactured a triangular structure by assembling three pairs of symmetric double-spirals (Fig. 3a). This structure was developed to be used as a three-jaw enveloping gripper. Owing to the high reversible extensibility of the double-spirals, the area inside the gripper (i.e., the area enclosed by the double-spirals) can be remarkably increased. Specifically, the triangular area inside the gripper, initially with a side length of 30 mm, expands into a triangle with a side length of 150 mm when the double-spirals are uncoiled (Fig. 3b). This expansion increases the area enclosed by the gripper up to 100 times its initial size, and upon release, the double-spiral gripper returns to its original coiled state (Video S2a). The inherent compliance of the double-spirals enables the gripper to adapt to objects of various shapes, while their reversible behavior allows the gripper to generate the required normal force for static friction with objects, developing a stable grip.

To further investigate the performance of the designed gripper, we 3D printed two double-spirals and quantified their loading–unloading behavior through tensile tests (Fig. 3c). We fixed one side of each double-spiral and used our uniaxial testing machine to apply specific displacements to the other side (i.e., 40, 80, and 120 mm). Given the initial distance of 30 mm between the two sides of the double-spiral, these applied displacements resulted in extended lengths of 70, 110, and 150 mm, respectively. After reaching each target length, we unloaded the double-spirals to 80% of the applied displacement (i.e., reducing them to 32, 64, and 96 mm, respectively) and held them at these positions for 60 s, a duration chosen to allow the material to undergo relaxation^51,52^. In all tests, the loading and unloading speeds were maintained at 1 mm/s, with equal closing and opening times observed across all cases. This process was repeated 30 times. In this experiment, the uncoiling process corresponds to the gripper being extended up to a specific area relative to the size of the object. Once extended, it is then released to grip the object. The gripper must maintain the position to securely hold the object; thereafter, it can extend once again to release the object.

The results show that: (i) an initial reduction in force values occurs over the first few cycles, a behavior commonly associated with the Mullins’ effect observed in elastomers, including TPU^51,52^. Following this initial phase of softening, the force values stabilize and display consistent behavior across subsequent cycles, as expected. (ii) The force required to keep the double-spirals extended in various positions initially rises during the 60-s holding period of each cycle and then stabilizes, with rather similar values observed across all three extension lengths. Holding the double-spiral at the 40, 80, and 120 mm positions results in approximately 15%, 14%, and 20% variations in force between the start and end of the holding period, respectively. Additionally, across all tests, the average holding force in the first cycle is 3.24 ± 0.3 N, decreases to 2.31 ± 0.2 N by the 15th cycle, and approaches 2.13 ± 0.2 N in the final cycle.

In the last step, we tested the functionality of the gripper and used it to grip four objects of different shapes and masses, from 200 to 750 g (Fig. 3d, Video S2b). It is shown here how the three-jaw gripper can envelop the objects and adapt to their shape. Furthermore, the experiment demonstrates that by taking advantage of friction force, the gripper can overcome the weight of objects and hold them.

### Mechanical interlocking structure

**Fig. 4:**
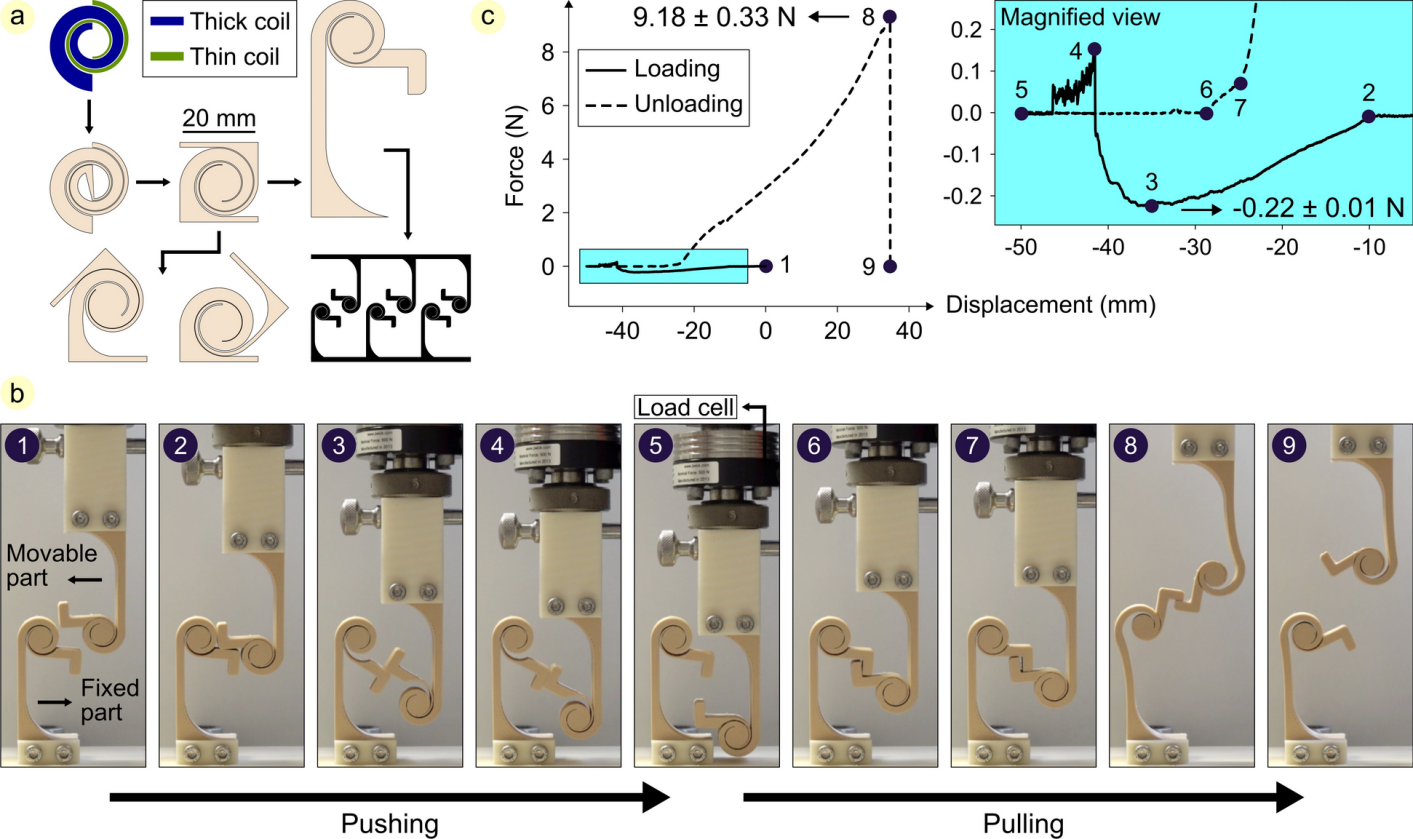
Mechanical interlocking structure. (**a**) Developing a double-spiral consisting of a thin and a thick spiral coil with low and high stiffness, respectively. The asymmetric rotation of this double-spiral is used to design a mechanical interlocking structure. (**b**) 3D printing and testing the performance of the developed interlocking structure. (**c**) The average force–displacement graph from experiments, with a magnified view of the loading-to-unloading transition phase, illustrating the anisotropic behavior of the structure in two different directions.

Herein, a double-spiral with asymmetric behavior in rotation is designed. This double-spiral consists of two spiral coils with different design variables and geometries: a thin and a thick spiral with low and high stiffness, respectively (Fig. 4a). We used this feature of the designed double-spiral to develop a mechanical interlocking structure. To characterize the performance of the structure, we manufactured it in two parts. One part was fixed, while the other, designated as the movable part, was subjected to a compressive and then a tensile force to measure the force required for their attachment and detachment, respectively (Fig. 4b, Video S3). The movable part was attached to a load cell, which was mounted on a platform connected to a linear stage, allowing precise measurement of both force and displacement. After resetting the movable part to its initial position before each trial, we performed the experiment three times and calculated the results as mean ± standard deviation (Fig. 4c).

The results show that the pulling force is more than 40 times the pushing force. By adjusting the design variables of two spiral coils forming the compliant double-spiral, anisotropic behavior of the structure can be tuned. This characteristic facilitates the development of tunable and reversible attachment. In addition to a single interlocking mechanism (consisting of two double-spiral parts), a set of double-spiral interlocking structures arranged rationally on two plates can be used to achieve a non-uniform attachment-detachment system with pre-programmed mechanical behavior. Probabilistic fasteners, frictional attachment pads, and temporary coupling mechanisms are examples of reversible attachment systems found in nature, which have been inspiring for the development of engineering designs beneficial for applications where quick and controlled attachment and detachment are required^60–62^.

### Adaptive energy-dissipative structure

The simple adjustability of the double-spiral geometry and its predictable non-linear mechanical behavior were used here to design an adaptive energy-dissipative structure. Our aim was to evaluate the ability of double-spirals with distinct geometries to dissipate energy through their high extensibility in response to different force values.

**Fig. 5:**
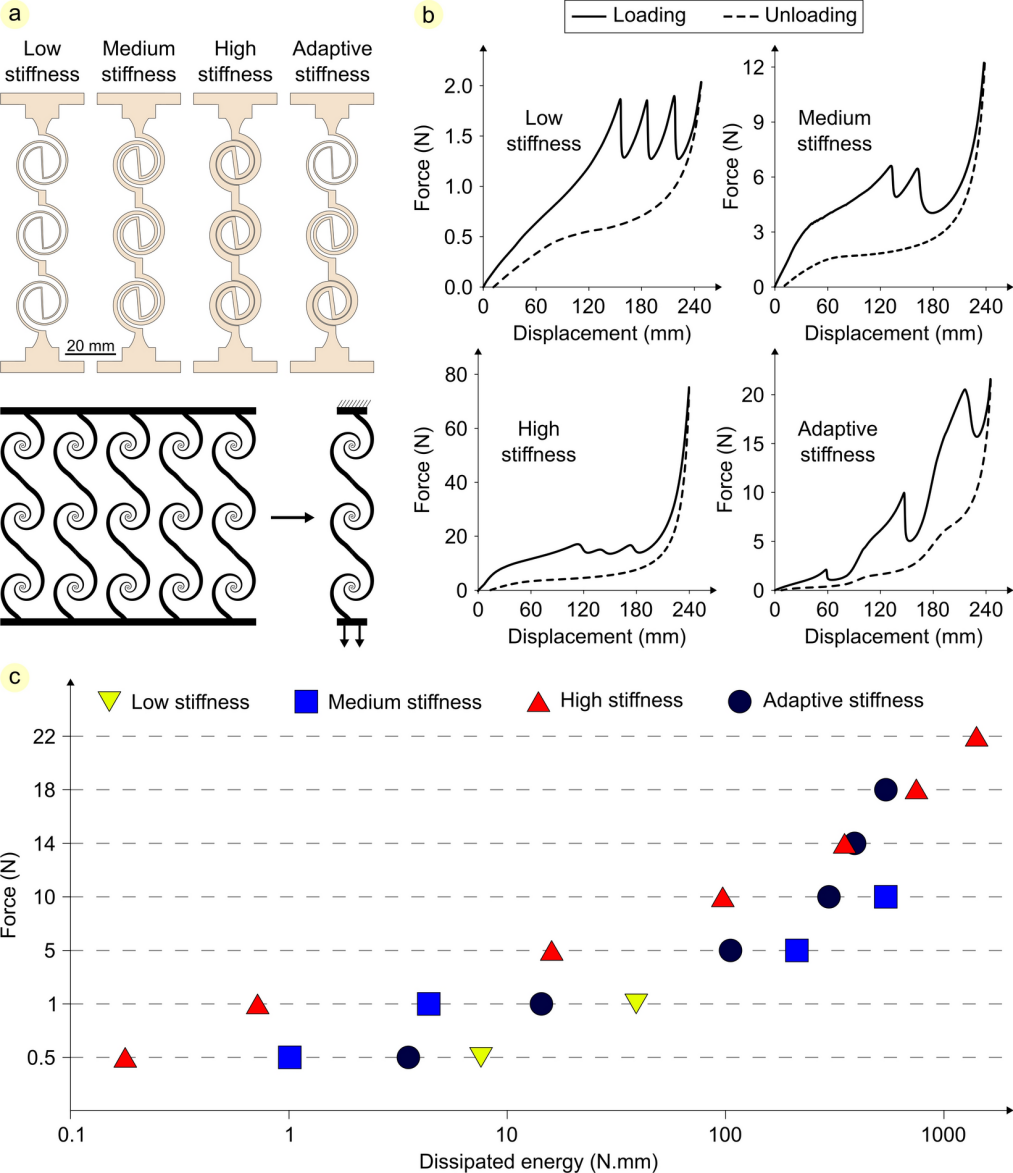
Adaptive energy-dissipative structure. (**a**) Using three double-spirals with different thicknesses to develop low, medium, high, and adaptive stiffness energy-dissipative structures. (**b**) Force–displacement graphs resulted from experimental tensile tests conducted on the four developed structures at a rate of 1 mm/s. (**c**) Comparing the amount of dissipated energy resulting from the deformation of four developed structures when subjected to the same tensile forces at a rate of 1 mm/s.

We developed three double-spirals with different initial thicknesses (i.e., 1, 2, and 3 mm). Three double-spirals with the smallest thickness (i.e., 1 mm) were connected to form a low stiffness structure (Fig. 5a). By connecting three double-spirals with 2 mm thickness and then three double-spirals with 3 mm thickness, we made medium and high stiffness structures, respectively. We also developed the structure with adaptive stiffness by connecting three double-spirals, each with a different thickness. Regarding the function of the developed structures, we investigated their mechanical behavior when subjected to tensile loading. Each model was extended to its maximum length (245 mm) at a rate of 1 mm/s and then unloaded at the same rate to observe its loading–unloading behavior (Fig. 5b, Fig. S1).

The energy-dissipative performance of the four developed structures was compared under the same tensile forces (Fig. S2). The area between the loading and unloading force–displacement curves was calculated to represent the amount of energy dissipated by each structure due to deformation in response to a specific force value (Fig. 5c, Table S3). The results show that the low, medium, and high stiffness structures are highly efficient only when subjected to a limited range of forces. In contrast, the adaptive stiffness structure, composed of double-spirals with distinct thicknesses, deforms under a wide variety of force values and consequently dissipates a considerable amount of energy.

Furthermore, to investigate the impact of loading rate on the mechanical behavior of the developed energy-dissipative structures, we conducted the same tensile tests on them at a rate of 10 mm/s and quantified the resulting energy dissipation (Fig. S3, Table S3). Although the values of displacement and dissipated energy vary with the loading rate, comparing the performance of structures leads to the same conclusion: The adaptive stiffness structure extends in response to a large variety of force values and dissipates more energy.

### Compliant planar joint

**Fig. 6:**
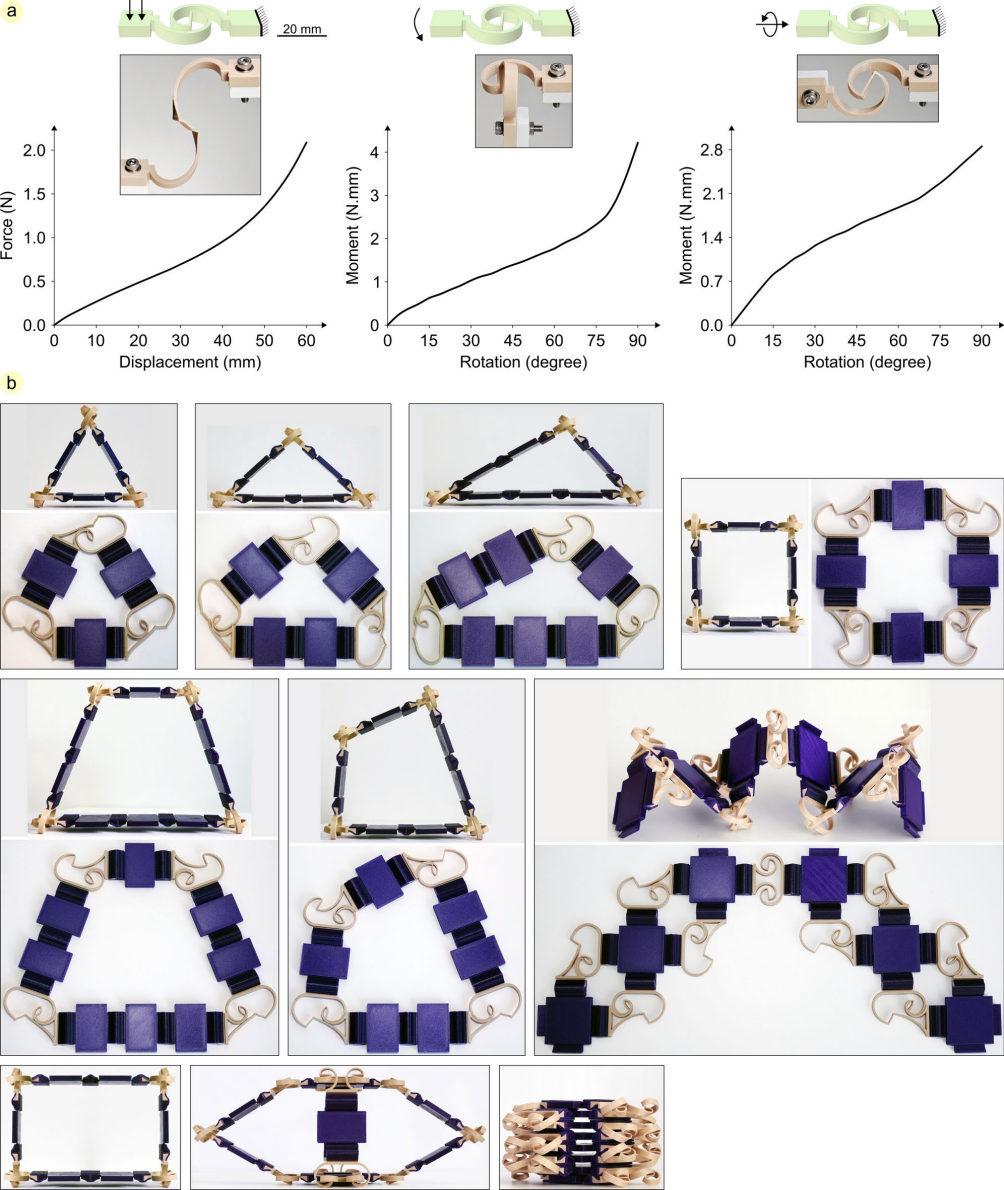
Compliant planar joint. (**a**) Quantifying the mechanical behavior of the 3D printed double-spiral under out-of-plane sliding, rotation, and torsion. (**b**) Using compliant double-spiral joints and stiff plates to make different modular structures. The developed structures can be easily deformed and switched between 3D spatial and planar states.

Double-spiral is used here as a monolithic compliant joint which allows in- and out-of-plane displacement and rotation of interconnected parts. Taking advantage of the fixed cross-sectional profile of double-spiral, its extrusion height can be simply tuned to meet our requirements. We designed a double-spiral with 5 mm extrusion height. This is a small extrusion height to facilitate out-of-plane deformation of the double-spiral. We then 3D printed two specimens to quantify their mechanical behavior when subjected to out-of-plane sliding, rotation, and torsion. We tested each specimen three times and presented the average force–displacement graphs (Fig. 6a). We then used the double-spirals as compliant joints to connect stiff plates to each other and developed modular structures with different shapes and mechanical behavior (Fig. 6b, Video S4).

Numerous structures can be developed by assembling different numbers of 3D printed double-spiral joints and plates. Desired shapes and deformation patterns are exploitable by reconfiguring the arrangement of the constituent modules. The high deformability of the double-spiral joints enables us to easily fold/unfold the plates and combine them in different spatial orientations. Moreover, the assembled structures are bistable, and we can easily switch them from a 3D spatial to a planar state. Similar to the mechanical behavior of double-spiral in response to planar loading scenarios that was studied before^14,18^, design variables can be used to manipulate the out-of-plane behavior of the joints. By assembling rationally designed double-spiral modules with specific spatial arrangements, desired properties are achievable.

## Discussion

Nowadays, the increased accessibility of advanced equipment and machines across various fields has significantly accelerated scientific and technological progress. Consequently, traditional engineering solutions are frequently rendered obsolete as technological advances raise further requirements. Many conventional materials and structures fail to meet the demands of modern systems, necessitating the replacement of outdated designs with novel and innovative alternatives. Modern structures have higher efficiency, durability, and unprecedented tunable properties. These novel structures possess the ability to adjust stiffness, change shape, adapt to different conditions, and perform multiple tasks or functions. Therefore, in many technological applications, they offer advantages over conventional materials and structures, which possess fixed mechanical properties tailored for limited tasks. Hence, efforts should be focused on promoting low-cost and easily accessible solutions for the development of intelligent and adaptive engineering structures^63–65^.

Double-spirals serve as an effective example of adaptive structures, which require no active actuation to achieve adaptability. Instead, they passively respond to their environment through an intelligence embedded in their design, known as Mechanical Intelligence^64,65^. In this study, we first developed a modeling software, called Double-Spiral Design, as a tool that facilitates the design of double-spirals with diverse geometries. The software enables users to design double-spirals consisting of four different either logarithmic or Archimedean spiral curves, to have a higher control over the geometry of their designs and achieve desired mechanical behavior, as previous studies have indicated the significant influence of the design variables on the mechanical behavior of double-spirals^14,18^. We then used double-spirals in the development of different structures and investigated their performance in practice to demonstrate the possibility of adopting the double-spiral as a mechanical element to passively regulate the functionality of structures, providing properties suitable for a variety of applications.

The two developed grippers (i.e., the freeform passive gripper and the highly extensible enveloping gripper) are both designed with the aim of reducing energy consumption during the process of grasping and holding objects by minimizing the need for active external actuators. In the freeform passive gripper, large conformability with objects of various shapes is achieved passively due to the high rotational compliance of each double-spiral, while increased stiffness of the structure under tension improves its load-bearing capacity without requiring additional energy input. Moreover, the highly extensible enveloping gripper holds objects passively through friction, without requiring continuous external force. In contrast, a wide range of existing soft grippers, depending on their material and geometrical design, rely on various actuation technologies—such as pneumatic, vacuum, hydraulic, tendon-driven, electrically driven, and shape-memory alloys—for deformation, stiffness tuning, conforming to and holding objects^66–69^.

The presented spiral-based designs function through rational assemblies of multiple double-spiral modules. In particular, the planar double-spiral joints, with multiple in-plane and out-of-plane degrees of freedom, facilitate the development of foldable metastructures that can be assembled in various configurations and dimensions. These structures can easily switch between planar and 3D spatial states while exhibiting the desired mechanical behavior in different directions. This simple, quick, and cost-effective reconfigurability of spiral-based modular structures offers notable advantages in both fabrication and functionality, potentially making them more effective than structures with pre-designed monolithic geometries currently in use for similar functions^70–74^.

When designing structures, there are instances where conflicting objectives need to be met, necessitating the identification of a trade-off point. In this study, we address one of these contradictory scenarios with the introduction of a highly extensible enveloping gripper (section “Highly extensible enveloping gripper”). On one hand, a double-spiral comprising thinner coils offers greater compliance, enabling better adaptation to objects of various shapes and increasing the contact area. On the other hand, a double-spiral with thicker coils can exert more force on the gripped objects, which is crucial for establishing static friction between the gripper and the objects. An optimal compromise between such contradictory objectives can be reached in future studies utilizing evolutionary algorithms.

TPU is a material suitable for fabricating flexible components in various engineering applications, owing to its remarkable mechanical properties, including high elasticity and resilience^48^. Consisting of hard and soft segments, TPU has a two-phase microstructure and demonstrates strong hysteresis, rate- and time-dependent behavior, as well as softening and hardening when subjected to mechanical loads^51–53^. Beyond our initial findings on the performance of spiral-based structures fabricated with TPU under a limited number of loading and unloading cycles, a more comprehensive assessment of their behavior over an increased number of cycles would provide deeper insights into their functionality, reversibility, and stability for extended use.

**Fig. 7:**
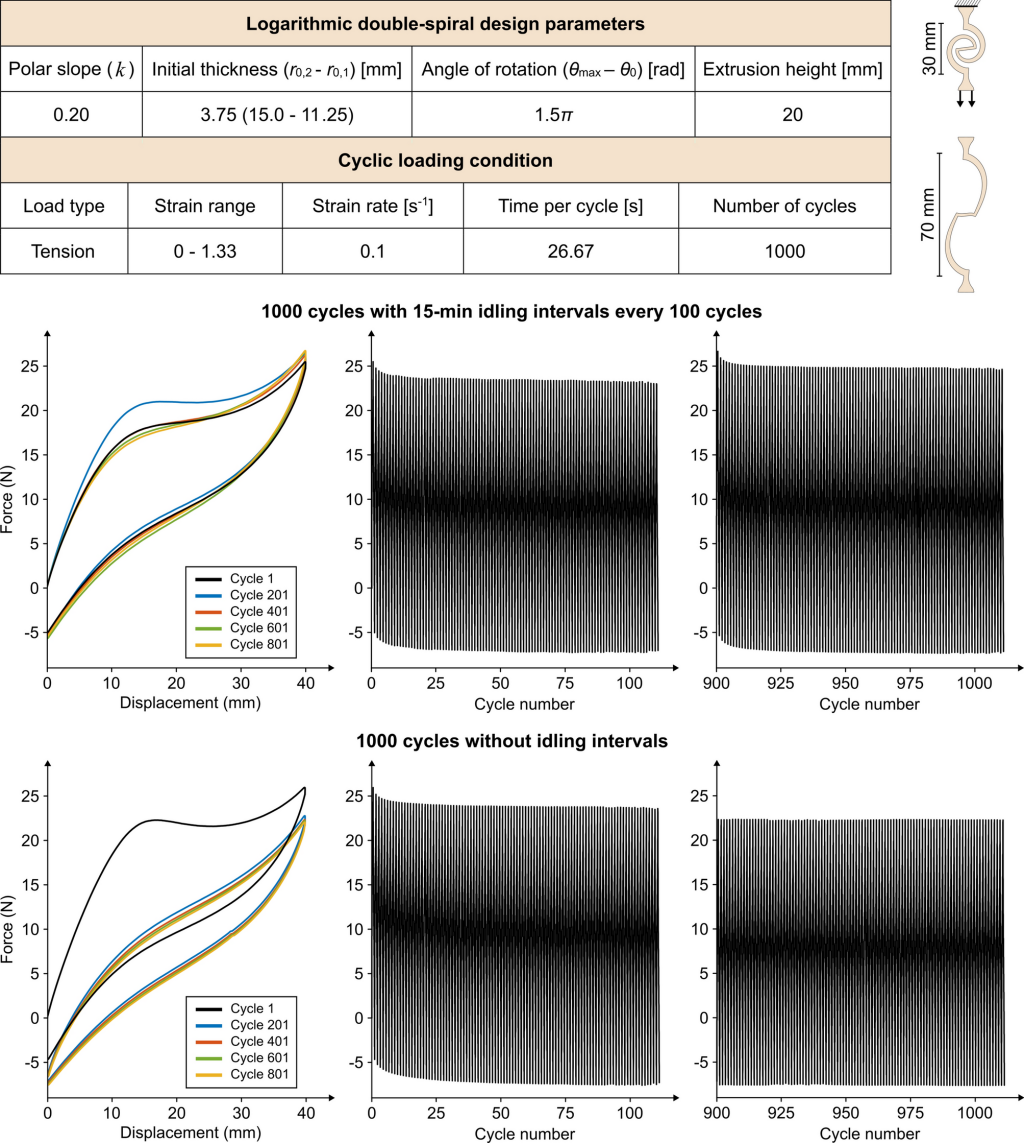
Investigating the mechanical behavior of double-spiral samples under 1000 cycles of tension with and without idling intervals. The design parameters of the logarithmic double-spiral utilized in the tests, along with the cyclic loading conditions, are provided in the table. Additionally, sketches of the double-spiral in its initial and deformed states are included. The force–displacement curves for both tests, with and without idling intervals, are presented to illustrate variations in the behavior of the double-spiral samples at the beginning of each 200 cycles. The force-cycle number plots capture the full range of cycles (1–100 and 901–1000), providing insights into the effects of 15-min idling intervals introduced every 100 cycles, as compared to continuous cycling without idling intervals.

Investigating the mechanical behavior of a sample double-spiral, subjected to 1000 cycles of uncoiling to the maximum length expected in its intended applications and then coiling back, reveals several noteworthy patterns (Fig. 7). First, an initial softening phase, characterized by a decrease in force over the first few cycles—a behavior well-known for TPU^51,52^. This is followed by the stabilization of force values, indicating the ability of double-spirals to maintain consistent performance under cyclic loading. With idling intervals, the double-spiral experiences slight hardening over time, exhibiting a modest recovery of force even in later cycles (900–1000), suggesting that rest periods allow for partial structural recovery and retention of stiffness^53^. In contrast, without idling, both stiffness and hysteresis gradually decrease, leading to a stable yet softened behavior over time.

These observations suggest that the double-spiral structure, even when subjected to extended cyclic loading, retains its functional integrity and exhibits stable mechanical performance, making it well-suited for applications requiring long-term durability and adaptability, such as in soft robotics. The cyclic loading tests, conducted at a high strain range and rate, were primarily designed to illustrate the overall performance of the double-spiral structure under continuous loading conditions. Further investigations could refine the loading parameters to align with specific functional requirements, as it is well-established that the range and rate of deformation, type of loading, and the volume fraction of soft and hard segments in the TPU can dramatically influence its mechanical behavior^49–53^.

Future studies can take advantage of other structural and material strategies to enhance the efficiency of double-spirals. (i) The mechanical interlocking structure developed in this study is an example showing how we can modify the geometry of a double-spiral to improve its functionality for a specific goal. (ii) The highly extensible enveloping gripper might grip objects better if a structural strategy is used to increase the adhesion or surface friction between the double-spirals and objects^57,75^. (iii) Since the contacts between the coils of double-spirals during deformation serve as a structural energy dissipation mechanism^76^—in addition to the strong energy dissipation arising from the microstructure of TPU^51,52^—the desired frictional behavior can be achieved by controlling the surface roughness of the coils through the fabrication process^77,78^. (iv) Regarding the equations of spirals (Eqs. 1 and 2), reducing the thickness of the coils of a double-spiral is a simple method to decrease its stiffness. However, manufacturing precision determines the minimum possible dimensions of parts. In such cases, porosity can be used as a strategy to control the mechanical behavior of a double-spiral^79^. (v) The fabrication of double-spirals using other materials, such as composite filaments composed of TPU and PLA^80,81^ or TPU and wood flour^82^ is another possibility that could be useful for the development of spiral-based structures with higher efficiency.

The main purpose of this paper was to showcase the versatility of compliant double-spirals as an element in various structures to attain specific mechanical characteristics. Detailed investigations using theoretical and experimental methods are required for each of the developed structures to explore optimized double-spirals and ensure predictability in their behavior. Furthermore, future studies could focus on comparing the performance of the developed spiral-based structures with other designs that are currently being used in engineering systems for similar purposes, providing a benchmark to better position spiral-based structures among alternative solutions.

## Conclusion

This study presents compliant double-spirals, inspired by highly deformable natural spirals, as versatile elements that embed mechanical intelligence within their design, enabling passive adaptability to environmental changes. This embedded intelligence empowers double-spirals to transmit the desired motion or force through reversible deformation and achieve essential functions—such as shape adaptation, stiffness tuning, and energy dissipation—making them suitable for applications in soft robotics, biomedical engineering, and flexible electronics. By developing the Double-Spiral Design software, we provide a tool for designing double-spirals with precise control over their geometry and mechanical behavior. Rationally designed double-spirals, arranged in specific configurations within engineering systems, can offer mechanical characteristics tailored to our particular needs. This research contributes to the exploration of new ideas and concepts surrounding double-spirals, paving the way for their use in developing innovative, adaptable, and versatile engineering structures.

## Supplementary Information

Supplementary Information 1.

Supplementary Information 2.

Supplementary Information 3.

Supplementary Video S1.

Supplementary Video S2a.

Supplementary Video S2b.

Supplementary Video S3.

Supplementary Video S4.

## Data Availability

All supporting data are made available either in the article or the electronic supplementary material.
