# Supplementary material for: Double-spiral as a bio-inspired functional element in engineering design
Source: Sci Rep. 2024 Nov 25;14:29225. doi: 10.1038/s41598-024-79630-6 (PMC11589583; doi:10.1038/s41598-024-79630-6)
Supplement: Supplementary file 1 — Supplementary Information 1. [file 41598_2024_79630_MOESM1_ESM.pdf]

Double-spiral as a bio-inspired functional element in engineering design

Mohsen Jafarpour[1], Mohammad Aryayi[2], Stanislav N. Gorb[1] & Hamed Rajabi[3, 4]

[1] Functional Morphology and Biomechanics, Institute of Zoology, Kiel University, 24118 Kiel, Germany

[2] University of Burgundy, 21000 Dijon, France

[3] Division of Mechanical Engineering and Design, School of Engineering, London South Bank University, London SE1 0AA, UK

[4] Mechanical Intelligence Research Group, School of Engineering, London South Bank University, London SE1 0AA, UK

Supplementary data

I) Double-Spiral Design software

Please download "Software.zip" and run the file "DSD.exe" to open the Double-Spiral Design software. Using this software you can develop your own double-spiral models.

II) Models

3D models of the spiral-based structures presented in this study.

III) Videos

Video S1. Freeform passive gripper being used for grasping objects of various shapes and masses. We added small magnets (Neodymium Rare Earth Magnets, 3×1.5 mm) to the outer surface of the double-spiral loop and created a magnetic gripper for picking up five random objects.

Video S2a. High reversible extension of the three-jaw enveloping gripper. The area inside the gripper can be remarkably increased, and when released, the double-spiral gripper returns to its original coiled state.

Video S2b. Highly extensible enveloping gripper being used for gripping objects of various shapes and masses.

Video S3. Mechanical behavior of the spiral-based interlocking structure. The results show that the pulling force is more than 40 times the pushing force.

Video S4. Deformation of the spiral-based modular structures under different loading scenarios. Double-spirals were used as compliant joints to develop modular structures with different shapes and mechanical behavior.

IV) Tables

Table S1. 3D printing settings.

Filament	Thermoplastic polyurethane (Flexfill TPU 98A)	Polylactic acid (PLA)
Produced by	Fillamentum addi(c)tive polymers, Czech Republic	Prusa Research, Praha, Czech Republic
Filament diameter (mm)	1.75	1.75
Nozzle diameter (mm)	0.4	0.4
Extrusion temperature (°C)	240	215
Bed temperature (°C)	50	60
Layer height (mm)	0.2	0.3
Fill pattern	Gyroid	Gyroid
Fill density (%)	20	50

Table S2. Design parameters of the logarithmic double-spirals which were employed in this study.

**Developed mechanical system**	**Design parameter**			
	Polar slope [k]	Initial thickness [$r_{0,2}$ - $r_{0,1}$] (mm)	Angle of rotation [$\theta_{max} - \theta_0$] (rad)	Extrusion height (mm)
Freeform passive gripper	0.20	1.5 (7.0-5.5)	1.5π	20
Highly extensible enveloping gripper	0.07	2.0 (15.0-13.0)	2.5π	40
Mechanical interlocking structure	0.10<br>0.10	1.4 (11.6-10.2)<br>4.8 (13.7-8.9)	3.0π<br>3.0π	20<br>20
Adaptive energy-dissipative structure	0.10	1.0 (12.0-11.0)	2.0π	20
	0.10	2.0 (12.0-10.0)	2.0π	20
	0.10	3.0 (12.0-9.0)	2.0π	20
Compliant planar joint	0.20	2.5 (15.0-12.5)	1.5π	5

Table S3. Extension of the low, medium, high, and adaptive stiffness structures under tensile forces, along with the corresponding energy dissipation due to their deformation.

**Loading rate: 1 mm/s**								
**Measured parameter**	**Developed structure**	**Applied force (N)**						
		0.5	1.0	5.0	10.0	14.0	18.0	22.0
Extension (mm)	Low stiffness	39.37	96.49					
	Medium stiffness	5.74	12.20	107.68	238.70			
	High stiffness	1.00	2.04	9.38	24.40	60.45	105.68	213.73
	Adaptive stiffness	19.13	43.74	115.50	178.68	190.34	208.09	
Dissipated energy (N.mm)	Low stiffness	7.67	39.00					
	Medium stiffness	1.02	4.41	210.85	543.73			
	High stiffness	0.18	0.72	16.12	96.54	362.77	750.17	1403.28
	Adaptive stiffness	3.57	14.38	107.38	298.15	380.16	547.74	
**Loading rate: 10 mm/s**								
**Measured parameter**	**Developed structure**	**Applied force (N)**						
		0.5	1.0	5.0	10.0	14.0	18.0	22.0
Extension (mm)	Low stiffness	30.25	72.46					
	Medium stiffness	4.20	8.09	51.03	232.41			
	High stiffness	1.35	2.15	7.65	16.75	31.22	65.13	100.29
	Adaptive stiffness	12.52	28.79	100.60	139.33	181.71	191.19	
Dissipated energy (N.mm)	Low stiffness	5.58	27.56					
	Medium stiffness	0.81	2.88	96.24	605.41			
	High stiffness	0.34	0.87	12.27	59.41	165.53	464.32	811.31
	Adaptive stiffness	2.23	10.45	88.62	249.80	379.14	456.13	

V) Figures

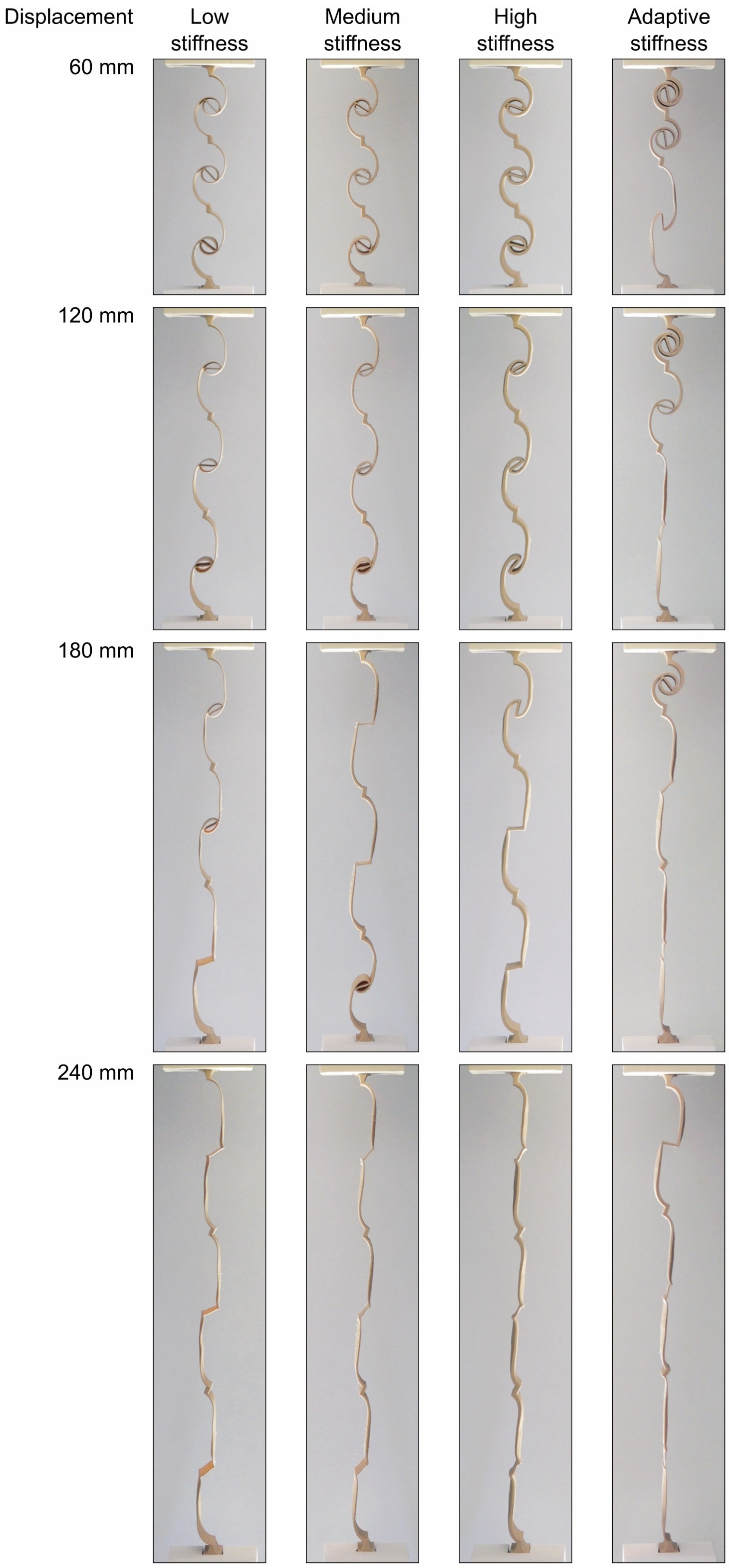

Fig S1. Tensile behavior of four structures consisted of geometrically different double-spirals. The figure shows the deformation of the low, medium, high, and adaptive stiffness structures, each extended to the same lengths.

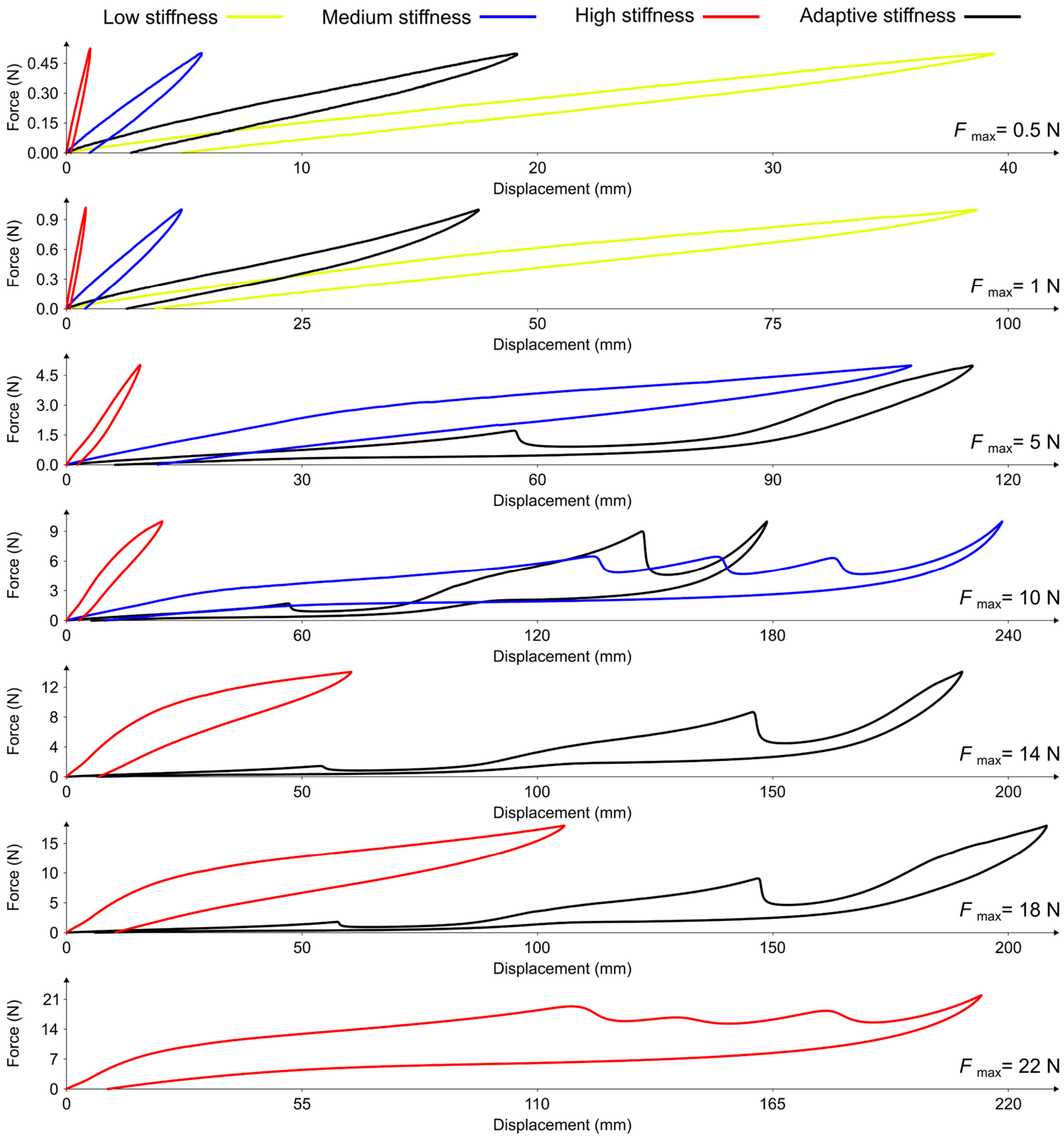

Fig S2. Mechanical behavior of the low, medium, high, and adaptive stiffness structures under tension. Force–displacement curves illustrate the hysteresis observed in the non-linear behavior of compliant double-spirals when subjected to tensile forces at a rate of 1 mm/s.

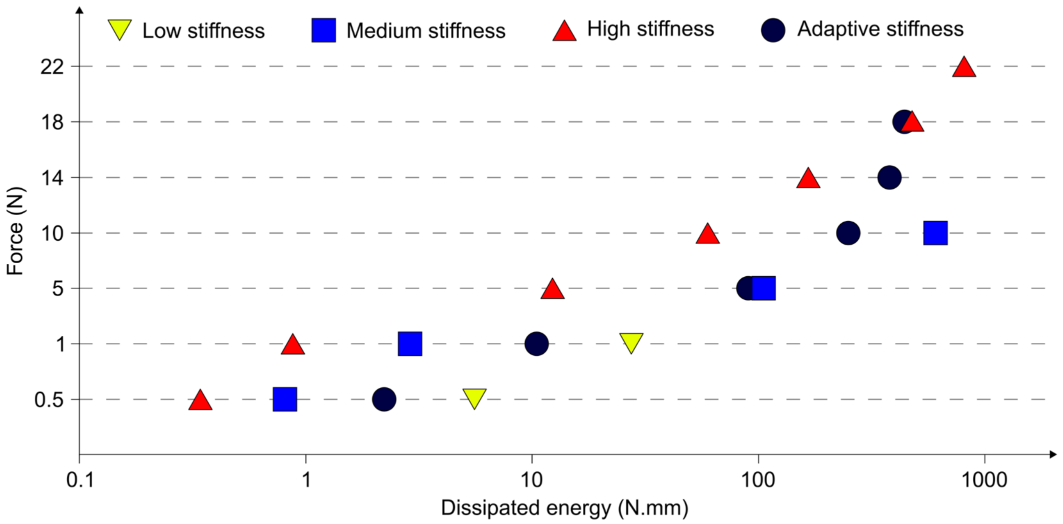

Fig S3. Comparing the amount of dissipated energy resulting from the deformation of four developed structures with low, medium, high, and adaptive stiffness, when subjected to the same tensile forces at a rate of 10 mm/s.
