# Supplementary figures and images for: Double-spiral as a bio-inspired functional element in engineering design
Source: Sci Rep. 2024 Nov 25;14:29225. doi: 10.1038/s41598-024-79630-6 (PMC11589583; doi:10.1038/s41598-024-79630-6)

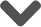

Supplement: Supplementary file 2 — Supplementary Information 2. [file 41598_2024_79630_MOESM2_ESM.zip › Software/stylesheets/1downarrow.png]

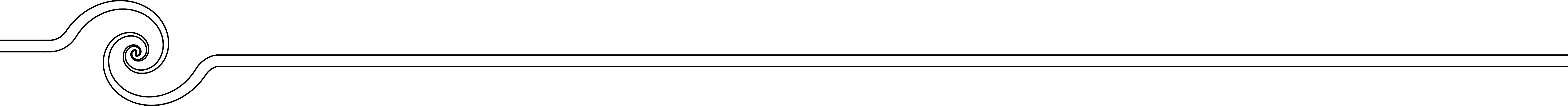

Supplement: Supplementary file 2 — Supplementary Information 2. [file 41598_2024_79630_MOESM2_ESM.zip › Software/stylesheets/banner.png]

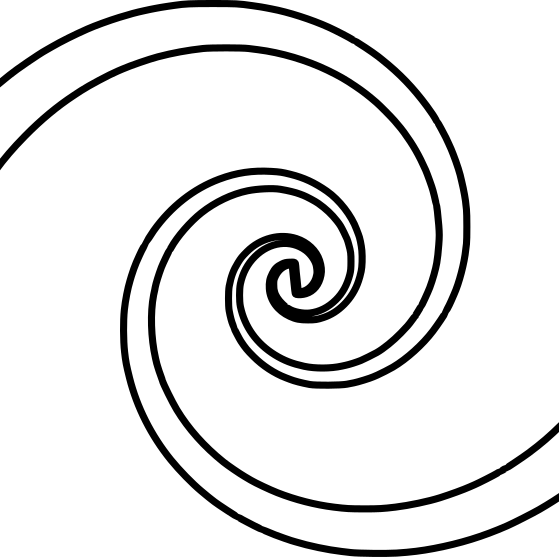

Supplement: Supplementary file 2 — Supplementary Information 2. [file 41598_2024_79630_MOESM2_ESM.zip › Software/stylesheets/logo.png]

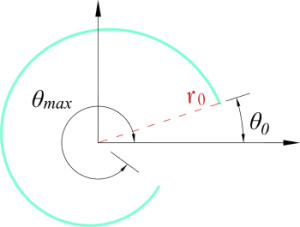

Supplement: Supplementary file 2 — Supplementary Information 2. [file 41598_2024_79630_MOESM2_ESM.zip › Software/stylesheets/spiral1.png]

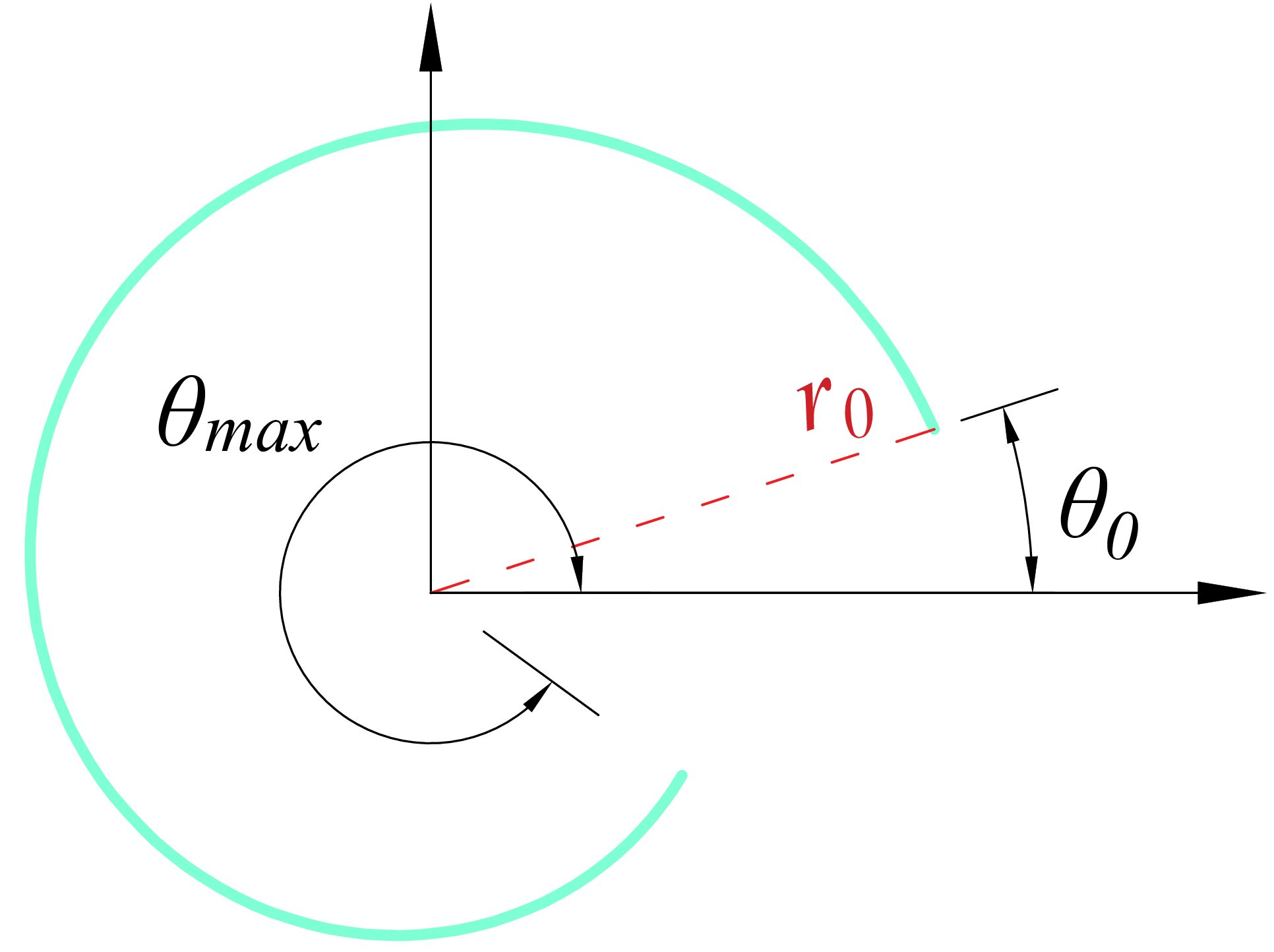

Supplement: Supplementary file 2 — Supplementary Information 2. [file 41598_2024_79630_MOESM2_ESM.zip › Software/stylesheets/spiral111.png]

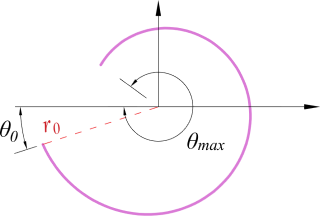

Supplement: Supplementary file 2 — Supplementary Information 2. [file 41598_2024_79630_MOESM2_ESM.zip › Software/stylesheets/spiral3.png]

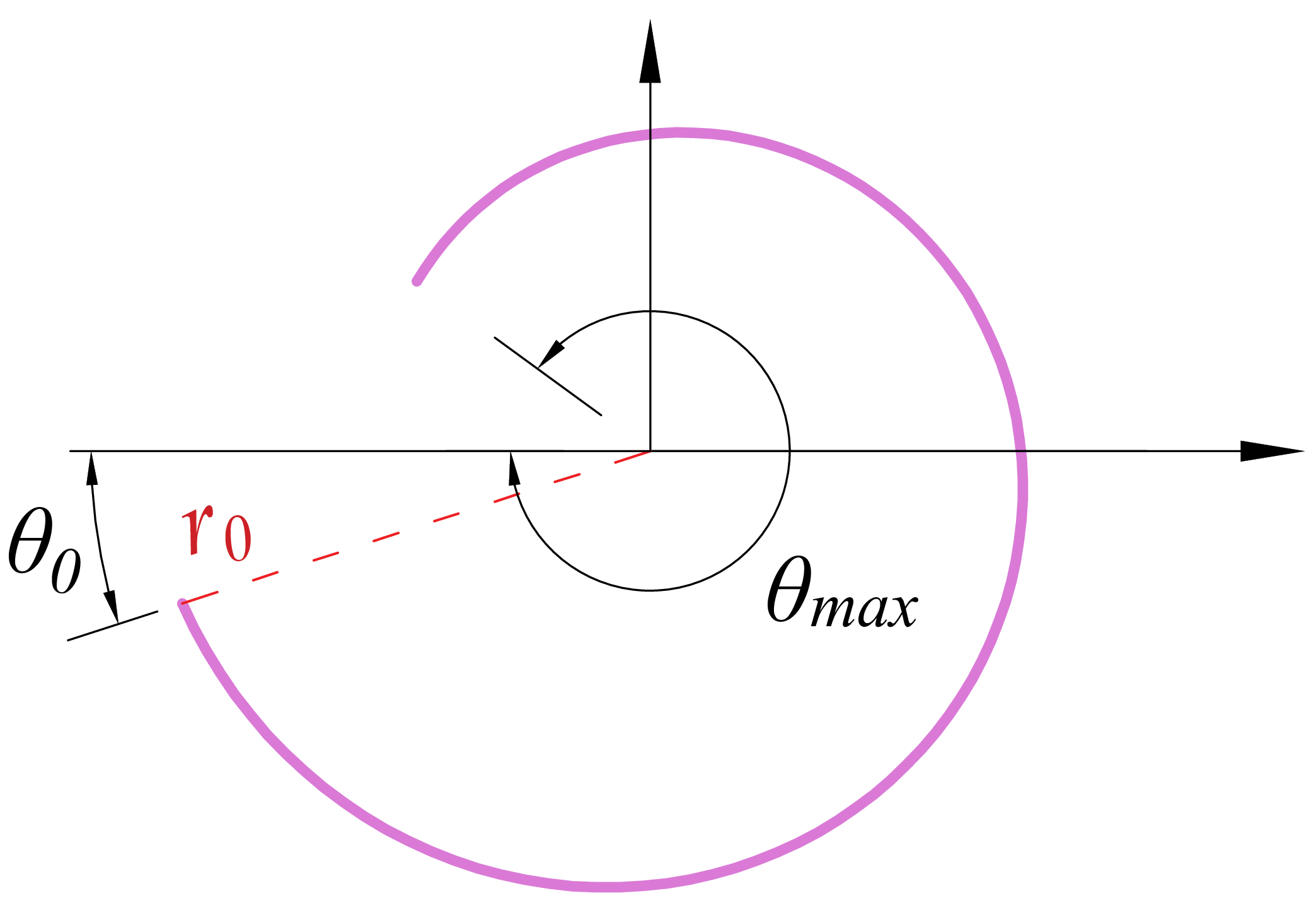

Supplement: Supplementary file 2 — Supplementary Information 2. [file 41598_2024_79630_MOESM2_ESM.zip › Software/stylesheets/spiral5443.png]

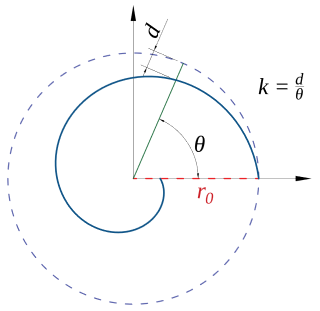

Supplement: Supplementary file 2 — Supplementary Information 2. [file 41598_2024_79630_MOESM2_ESM.zip › Software/stylesheets/spiralKarch.png]

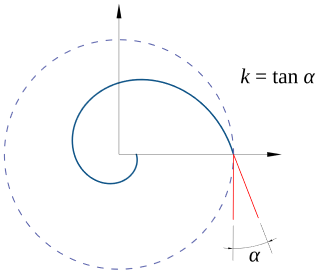

Supplement: Supplementary file 2 — Supplementary Information 2. [file 41598_2024_79630_MOESM2_ESM.zip › Software/stylesheets/spiralKLog.png]

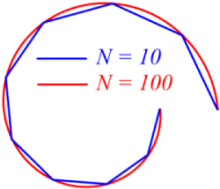

Supplement: Supplementary file 2 — Supplementary Information 2. [file 41598_2024_79630_MOESM2_ESM.zip › Software/stylesheets/spiralN.png]
